# Variations in seasonal solar insolation are associated with a history of suicide attempts in bipolar I disorder

**DOI:** 10.1186/s40345-021-00231-7

**Published:** 2021-09-01

**Authors:** Michael Bauer, Tasha Glenn, Eric D. Achtyes, Martin Alda, Esen Agaoglu, Kürşat Altınbaş, Ole A. Andreassen, Elias Angelopoulos, Raffaella Ardau, Edgar Arrua Vares, Memduha Aydin, Yavuz Ayhan, Christopher Baethge, Rita Bauer, Bernhard T. Baune, Ceylan Balaban, Claudia Becerra-Palars, Aniruddh P. Behere, Prakash B. Behere, Habte Belete, Tilahun Belete, Gabriel Okawa Belizario, Frank Bellivier, Robert H. Belmaker, Francesco Benedetti, Michael Berk, Yuly Bersudsky, Şule Bicakci, Harriet Birabwa-Oketcho, Thomas D. Bjella, Conan Brady, Jorge Cabrera, Marco Cappucciati, Angela Marianne Paredes Castro, Wei-Ling Chen, Eric Y. Wo Cheung, Silvia Chiesa, Marie Crowe, Alessandro Cuomo, Sara Dallaspezia, Maria Del Zompo, Pratikkumar Desai, Seetal Dodd, Markus Donix, Bruno Etain, Andrea Fagiolini, Frederike T. Fellendorf, Ewa Ferensztajn-Rochowiak, Jess G. Fiedorowicz, Kostas N. Fountoulakis, Mark A. Frye, Pierre A. Geoffroy, Ana Gonzalez-Pinto, John F. Gottlieb, Paul Grof, Bartholomeus C. M. Haarman, Hirohiko Harima, Mathias Hasse-Sousa, Chantal Henry, Lone Høffding, Josselin Houenou, Massimiliano Imbesi, Erkki T. Isometsä, Maja Ivkovic, Sven Janno, Simon Johnsen, Flávio Kapczinski, Gregory N. Karakatsoulis, Mathias Kardell, Lars Vedel Kessing, Seong Jae Kim, Barbara König, Timur L. Kot, Michael Koval, Mauricio Kunz, Beny Lafer, Mikael Landén, Erik R. Larsen, Melanie Lenger, Ute Lewitzka, Rasmus W. Licht, Carlos Lopez-Jaramillo, Alan MacKenzie, Helle Østergaard Madsen, Simone Alberte Kongstad A. Madsen, Jayant Mahadevan, Agustine Mahardika, Mirko Manchia, Wendy Marsh, Monica Martinez-Cengotitabengoa, Klaus Martiny, Yuki Mashima, Declan M. McLoughlin, Ybe Meesters, Ingrid Melle, Fátima Meza-Urzúa, Mok Yee Ming, Scott Monteith, Muthukumaran Moorthy, Gunnar Morken, Enrica Mosca, Anton A. Mozzhegorov, Rodrigo Munoz, Starlin V. Mythri, Fethi Nacef, Ravi K. Nadella, Takako Nakanotani, René Ernst Nielsen, Claire O‘Donovan, Adel Omrani, Yamima Osher, Uta Ouali, Maja Pantovic-Stefanovic, Pornjira Pariwatcharakul, Joanne Petite, Andrea Pfennig, Yolanda Pica Ruiz, Maximilian Pilhatsch, Marco Pinna, Maurizio Pompili, Richard Porter, Danilo Quiroz, Francisco Diego Rabelo-da-Ponte, Raj Ramesar, Natalie Rasgon, Woraphat Ratta-apha, Michaela Ratzenhofer, Maria Redahan, M. S. Reddy, Andreas Reif, Eva Z. Reininghaus, Jenny Gringer Richards, Philipp Ritter, Janusz K. Rybakowski, Leela Sathyaputri, Ângela M. Scippa, Christian Simhandl, Emanuel Severus, Daniel Smith, José Smith, Paul W. Stackhouse, Dan J. Stein, Kellen Stilwell, Sergio Strejilevich, Kuan-Pin Su, Mythily Subramaniam, Ahmad Hatim Sulaiman, Kirsi Suominen, Andi J. Tanra, Yoshitaka Tatebayashi, Wen Lin Teh, Leonardo Tondo, Carla Torrent, Daniel Tuinstra, Takahito Uchida, Arne E. Vaaler, Julia Veeh, Eduard Vieta, Biju Viswanath, Maria Yoldi-Negrete, Oguz Kaan Yalcinkaya, Allan H. Young, Yosra Zgueb, Peter C. Whybrow

**Affiliations:** 1 Department of Psychiatry and Psychotherapy, Faculty of Medicine, University Hospital Carl Gustav Carus, Technische Universität Dresden, Dresden, Germany; 2 ChronoRecord Association, Fullerton, CA USA; 3 Division of Psychiatry and Behavioral Medicine, Michigan State University College of Human Medicine, Grand Rapids, MI USA; 4 Department of Psychiatry, Dalhousie University, Halifax, NS Canada; 5 Department of Psychiatry, Hacettepe University Faculty of Medicine, Ankara, Turkey; 6 Department of Psychiatry, Selcuk University Faculty of Medicine, Mazhar Osman Mood Center, Konya, Turkey; 7 NORMENT Centre, Division of Mental Health and Addiction, Oslo University Hospital and Institute of Clinical Medicine, University of Oslo, Oslo, Norway; 8 Department of Psychiatry, National and Capodistrian University of Athens, Medical School, Eginition Hospital, Athens, Greece; 9 Section of Neurosciences and Clinical Pharmacology, Department of Biomedical Sciences, University of Cagliari, Sardinia, Italy; 10 Department of Psychiatry, Selcuk University Faculty of Medicine, Konya, Turkey; 11 Department of Psychiatry and Psychotherapy, University of Cologne Medical School, Cologne, Germany; 12 Department of Psychiatry, University of Münster, Munster, Germany; 13 Department of Psychiatry, Melbourne Medical School, The University of Melbourne, Melbourne, Australia; 14 The Florey Institute of Neuroscience and Mental Health, The University of Melbourne, Parkville, VIC Australia; 15 Department of Psychiatry, Psychosomatic Medicine and Psychotherapy, University Hospital Frankfurt, Johann Wolfgang Goethe-Universität Frankfurt am Main, Frankfurt am Main, Germany; 16 National Institute of Psychiatry “Ramón de la Fuente Muñiz”, Mexico City, Mexico; 17 Child and Adolescent Psychiatry, Helen DeVos Children’s Hospital, Michigan State University-CHM, Grand Rapids, MI USA; 18 Department of Psychiatry, Jawaharlal Nehru Medical College, Datta Meghe Institute of Medical Sciences (Deemed University), Wardha, India; 19 Department of Psychiatry, College of Medicine and Health Sciences, Bahir Dar University, Bahir Dar, Ethiopia; 20 Bipolar Disorder Research Program, Department of Psychiatry, University of São Paulo Medical School, São Paulo, Brazil; 21 Département de Psychiatrie et de Médecine Addictologique, Assistance Publique-Hôpitaux de Paris, INSERM UMR-S1144, Université de Paris, FondaMental Foundation, Paris, France; 22 Professor Emeritus of Psychiatry, Ben Gurion University of the Negev, Beer Sheva, Israel; 23 University Vita-Salute San Raffaele, Milan, Italy; 24 Psychiatry and Clinical Psychobiology, Division of Neuroscience, San Raffaele Scientific Institute, Milan, Italy; 25 Deakin University, IMPACT-The Institute for Mental and Physical Health and Clinical Translation, School of Medicine, Barwon Health, Geelong, Australia; 26 Orygen, The National Centre of Excellence in Youth Mental Health, Centre for Youth Mental Health, Florey Institute for Neuroscience and Mental Health, Department of Psychiatry, The University of Melbourne, Melbourne, Australia; 27 Department of Psychiatry, Faculty of Health Sciences, Beer Sheva Mental Health Center, Ben Gurion University of the Negev, Beer Sheva, Israel; 28 Department of Psychiatry, Baskent University Faculty of Medicine, Ankara, Turkey; 29 Butabika Hospital, Kampala, Uganda; 30 Department of Psychiatry, Trinity College Dublin, St Patrick’s University Hospital, Dublin, Ireland; 31 Mood Disorders Clinic, Dr. Jose Horwitz Psychiatric Institute, Santiago de Chile, Chile; 32 Department of Mental Health and Substance Abuse, Piacenza, Italy; 33 Department of Psychiatry, Chiayi Branch, Taichung Veterans General Hospital, Chiayi, Taiwan; 34 Private Practice, Central, Hong Kong; 35 Department of Psychological Medicine, University of Otago, Christchurch, New Zealand; 36 Department of Molecular Medicine, University of Siena School of Medicine, Siena, Italy; 37 Pine Rest Christian Mental Health Services, Grand Rapids, MI USA; 38 Department of Psychiatry, University of Melbourne, Parkville, VIC Australia; 39 Department of Psychiatry and Psychotherapeutic Medicine, Medical University Graz, Graz, Austria; 40 Department of Adult Psychiatry, Poznan University of Medical Sciences, Poznan, Poland; 41 Department of Psychiatry, School of Epidemiology and Public Health, University of Ottawa, Ottawa, ON Canada; 42 3rd Department of Psychiatry, School of Medicine, Faculty of Health Sciences, Aristotle University of Thessaloniki, Thessaloniki, Greece; 43 Department of Psychiatry and Psychology, Mayo Clinic Depression Center, Mayo Clinic, Rochester, MN USA; 44 Département de Psychiatrie et d’addictologie, AP-HP, Hopital Bichat-Claude Bernard, Paris, France; 45 GHU Paris-Psychiatry and Neurosciences, 75014 Paris, France; 46 Université de Paris, NeuroDiderot, Inserm, Paris, France; 47 BIOARABA, Department of Psychiatry, University Hospital of Alava, University of the Basque Country, CIBERSAM, Vitoria, Spain; 48 Department of Psychiatry, Feinberg School of Medicine, Northwestern University, Chicago, IL USA; 49 Mood Disorders Center of Ottawa and the Department of Psychiatry, University of Toronto, Ottawa, Canada; 50 Department of Psychiatry, University Medical Center Groningen, University of Groningen, Groningen, The Netherlands; 51 Department of Psychiatry, Tokyo Metropolitan Matsuzawa Hospital, Setagaya, Tokyo Japan; 52 Department of Psychiatry, Universidade Federal do Rio Grande do Sul, Porto Alegre, Brazil; 53 Department of Psychiatry, GHU Paris Psychiatrie & Neurosciences, 75014 Paris, France; 54 Université de Paris, 75006 Paris, France; 55 Department of Clinical Research, University of Southern Denmark, Odense, Denmark; 56 Université Paris Est Créteil, INSERM, IMRB, Translational Neuropsychiatry, Fondation FondaMental, 94010 Créteil, France; 57 Université Paris Saclay, CEA, Neurospin, 91191 Gif-sur-Yvette, France; 58 Department of Psychiatry, University of Helsinki and Helsinki University Hospital, Helsinki, Finland; 59 National Institute for Health and Welfare, Helsinki, Finland; 60 University Clinical Center of Serbia, Clinic for Psychiatry, Belgrade, Serbia; 61 Department of Psychiatry, University of Tartu, Tartu, Estonia; 62 Unit for Psychiatric Research, Aalborg University Hospital, Aalborg, Denmark; 63 Department of Psychiatry and Neurochemistry, Institute of Neuroscience and Physiology, The Sahlgrenska Academy, University of Gothenburg, Gothenburg, Sweden; 64 Copenhagen Affective Disorder Research Center (CADIC), Psychiatric Center Copenhagen, Rigshospitalet, Copenhagen, Denmark; 65 Department of Psychiatry, Cheongju Hospital, Cheongju, South Korea; 66 BIPOLAR Zentrum Wiener Neustadt, Wiener Neustadt, Austria; 67 Khanty-Mansiysk Clinical Psychoneurological Hospital, Khanty-Mansiysk, Russia; 68 Department of Neuroscience, Michigan State University, East Lansing, MI USA; 69 Department of Medical Epidemiology and Biostatistics, Karolinska Institutet, Stockholm, Sweden; 70 Mental Health Department Odense, University Clinic and Department of Regional Health Research, University of Southern Denmark, Esbjerg, Denmark; 71 Psychiatry, Aalborg University Hospital, Aalborg, Denmark; 72 Department of Clinical Medicine, Aalborg University, Aalborg, Denmark; 73 Mood Disorders Program, Hospital Universitario San Vicente Fundación, Research Group in Psychiatry, Department of Psychiatry, Faculty of Medicine, Universidad de Antioquia, Medellín, Colombia; 74 Forensic Psychiatry, University of Glasgow, NHS Greater Glasgow and Clyde, Glasgow, UK; 75 Copenhagen University Hospitals, Psychiatric Centre Copenhagen, Copenhagen, Denmark; 76 Department of Psychiatry, National Institute of Mental Health and Neuro Sciences (NIMHANS), Bengaluru, India; 77 Department of Psychiatry, Faculty of Medicine, Mataram University, Mataram, Indonesia; 78 Department of Pharmacology, Dalhousie University, Halifax, NS Canada; 79 Section of Psychiatry, Department of Medical Science and Public Health, University of Cagliari, Cagliari, Italy; 80 Unit of Clinical Psychiatry, University Hospital Agency of Cagliari, Cagliari, Italy; 81 Department of Psychiatry, University of Massachusetts Medical School, Worcester, MA USA; 82 Osakidetza, Basque Health Service, BioAraba Research Institute, University of the Basque Country, Vitoria, Spain; 83 Department of Neuropsychiatry, Keio University School of Medicine, Tokyo, Japan; 84 Department of Psychiatry, Trinity College Institute of Neuroscience, Trinity College Dublin, St Patrick’s University Hospital, Dublin, Ireland; 85 Department of General Psychiatry, Mood Disorders Unit, Institute of Mental Health, Singapore City, Singapore; 86 Michigan State University College of Human Medicine, Traverse City Campus, Traverse City, MI USA; 87 Department of Mental Health, Norwegian University of Science and Technology-NTNU, Trondheim, Norway; 88 Department of Psychiatry, St Olavs’ University Hospital, Trondheim, Norway; 89 Soviet Psychoneurological Hospital, Urai, Russia; 90 Department of Psychiatry, University of California San Diego, San Diego, CA USA; 91 Asha Bipolar Clinic, Asha Hospital, Hyderabad, Telangana India; 92 Razi Hospital, Faculty of Medicine, University of Tunis-El Manar, Tunis, Tunisia; 93 Affective Disorders Research Project, Tokyo Metropolitan Institute of Medical Science, Setagaya, Tokyo Japan; 94 Tunisian Bipolar Forum, Érable Médical Cabinet 324, Lac 2, Tunis, Tunisia; 95 Department of Psychiatry, Faculty of Medicine, Siriraj Hospital, Mahidol University, Bangkok, Thailand; 96 Hospital “Ángeles del Pedregal”, Mexico City, Mexico; 97 Department of Psychiatry and Psychotherapy, Elblandklinikum Radebeul, Radebeul, Germany; 98 Lucio Bini Mood Disorder Center, Cagliari, Italy; 99 Department of Neurosciences, Mental Health and Sensory Organs, Sant’Andrea Hospital, Sapienza University of Rome, Rome, Italy; 100 Deparment of Psychiatry, Diego Portales University, Santiago de Chile, Chile; 101 SA MRC Genomic and Precision Medicine Research Unit, Division of Human Genetics, Department of Pathology, Institute of Infectious Diseases and Molecular Medicine, University of Cape Town, Cape Town, South Africa; 102 Department of Psychiatry and Behavioral Sciences, Stanford School of Medicine, Palo Alto, CA USA; 103 Departments of Psychiatry, Epidemiology, and Internal Medicine, Iowa Neuroscience Institute, The University of Iowa, Iowa City, IA USA; 104 Department of Neuroscience and Mental Health, Federal University of Bahia, Salvador, Brazil; 105 Bipolar Zentrum Wiener Neustadt, Sigmund Freud Privat Universität, Vienna, Austria; 106 Centre for Clinical Brain Sciences, University of Edinburgh, Edinburgh, Scotland, UK; 107 Bipolar Disorder Program, Neuroscience Institute, Favaloro University, Buenos Aires, Argentina; 108 Science Directorate/Climate Science Branch, NASA Langley Research Center, Hampton, VA USA; 109 Department of Psychiatry, MRC Unit On Risk and Resilience in Mental Disorders, University of Cape Town, Cape Town, South Africa; 110 College of Medicine, China Medical University (CMU), Taichung, Taiwan; 111 An-Nan Hospital, China Medical University, Tainan, Taiwan; 112 Research Division, Institute of Mental Health, Singapore, Singapore; 113 Department of Psychological Medicine, Faculty of Medicine, University of Malaya, Kuala Lumpur, Malaysia; 114 Department of Social Services and Health Care, Psychiatry, City of Helsinki, Helsinki, Finland; 115 Department of Psychiatry, Faculty of Medicine, Hasanuddin University, Makassar, Indonesia; 116 McLean Hospital-Harvard Medical School, Boston, MA USA; 117 Mood Disorder Lucio Bini Centers, Cagliari e Roma, Italy; 118 Clinical Institute of Neuroscience, Hospital Clinic, University of Barcelona, IDIBAPS, CIBERSAM, Barcelona, Catalonia Spain; 119 Subdirección de Investigaciones Clínicas, Instituto Nacional de Psiquiatría Ramón de la Fuente Muñíz, Mexico City, Mexico; 120 Department of Psychological Medicine, Institute of Psychiatry, Psychology and Neuroscience, King’s College London, London, UK; 121 Department of Psychiatry and Biobehavioral Sciences, Semel Institute for Neuroscience and Human Behavior, University of California Los Angeles (UCLA), Los Angeles, CA USA

**Keywords:** Bipolar disorder, Suicide, Sunlight, Solar insolation, Psychiatry, Circadian, Seasonal variation

## Abstract

**Background:**

Bipolar disorder is associated with circadian disruption and a high risk of suicidal behavior. In a previous exploratory study of patients with bipolar I disorder, we found that a history of suicide attempts was associated with differences between winter and summer levels of solar insolation. The purpose of this study was to confirm this finding using international data from 42% more collection sites and 25% more countries.

**Methods:**

Data analyzed were from 71 prior and new collection sites in 40 countries at a wide range of latitudes. The analysis included 4876 patients with bipolar I disorder, 45% more data than previously analyzed. Of the patients, 1496 (30.7%) had a history of suicide attempt. Solar insolation data, the amount of the sun’s electromagnetic energy striking the surface of the earth, was obtained for each onset location (479 locations in 64 countries).

**Results:**

This analysis confirmed the results of the exploratory study with the same best model and slightly better statistical significance. There was a significant inverse association between a history of suicide attempts and the ratio of mean winter insolation to mean summer insolation (mean winter insolation/mean summer insolation). This ratio is largest near the equator which has little change in solar insolation over the year, and smallest near the poles where the winter insolation is very small compared to the summer insolation. Other variables in the model associated with an increased risk of suicide attempts were a history of alcohol or substance abuse, female gender, and younger birth cohort. The winter/summer insolation ratio was also replaced with the ratio of minimum mean monthly insolation to the maximum mean monthly insolation to accommodate insolation patterns in the tropics, and nearly identical results were found. All estimated coefficients were significant at p < 0.01.

**Conclusion:**

A large change in solar insolation, both between winter and summer and between the minimum and maximum monthly values, may increase the risk of suicide attempts in bipolar I disorder. With frequent circadian rhythm dysfunction and suicidal behavior in bipolar disorder, greater understanding of the optimal roles of daylight and electric lighting in circadian entrainment is needed.

## Introduction

The risk for suicidal behavior for those with bipolar disorder is estimated to be 20–30 times higher than for the general population (Pompili et al. 2013; Shaffer 2015; Dong et al. 2019; Plans et al. 2019). Risk factors for suicidal behavior in bipolar disorder include depression, agitation, impulsivity, comorbid alcohol or substance abuse, prior suicidal acts, recent discharge from a psychiatric hospital, along with genetic, demographic, socioeconomic and cultural factors, and stressful life events (Pompili et al. 2013; Shaffer 2015; Tondo et al. 2021; Bachmann 2018; Plans et al. 2019; Tidemalm et al. 2014). Additionally, international epidemiology studies of the general population spanning several decades report seasonality in suicide attempts and deaths with a peak in spring or summer (Galvão et al. 2018; Woo et al. 2012; Su et al. 2020; Postolache et al. 2010; Oladunjoye et al. 2020; Christodoulou et al. 2012; Coimbra et al. 2016; Petridou et al. 2002).

There is increasing recognition of the profound and diverse impacts of daylight on human physiology and behavior, and the complexity of the mechanisms underlying the human response to light (Münch et al. 2017; Aranda and Schmidt 2021; Foster 2020). In addition to vision, daylight modulates circadian timing, the sleep–wake cycle, daily neuroendocrine functions, alertness, performance, mood and thermoregulation (Wirz-Justice et al. 2020; Paul and Brown 2019; Prayag et al. 2019; Cajochen 2007; Fisk et al. 2018; LeGates et al. 2014). Alterations in circadian rhythm are a major component of mood disorders (Logan and McClung 2019; Jones and Benca 2015; McClung 2013; Ketchesin et al. 2020), with disruptions in sleep, hormonal secretion, mood regulation and social rhythms occurring frequently in bipolar disorder (Melo et al. 2017; Takaesu 2018; McCarthy 2019; Gonzalez 2014). The effects of circadian disruptions in bipolar disorder are interrelated and can both trigger and exacerbate symptoms (Harvey 2008; Walker et al. 2020; Geoffroy 2018). About 25% of patients exhibit a seasonal pattern in the course of bipolar disorder (Geoffroy et al. 2014; Maruani et al. 2018).

In a prior exploratory study of patients with bipolar I disorder, we found that a history of suicide attempts was associated with living in locations with a large change in solar insolation between winter and summer (Bauer et al. 2019). Solar insolation (incoming solar radiation) is defined as the amount of electromagnetic energy from the sun striking a surface area on earth (Stackhouse et al. 2018). The aim of the current study was to investigate whether a repeat analysis with more data would confirm or contradict the results of the exploratory study. In addition, an analysis using the ratio of the minimum mean monthly insolation to the maximum mean monthly insolation was added to accommodate the insolation patterns in the tropics. The prior analysis included data from 50 collection sites in 32 countries. This analysis used 45% more data both from new and prior collection sites, including data from 71 collection sites in 40 countries with diverse cultures, healthcare systems, and climates.

## Methods

### Data collection

Data were collected by direct questioning, reviewing records, or both. All patients had a diagnosis of bipolar disorder from a psychiatrist according to DSM-IV or DSM-5 criteria. Study approval was obtained from local institutional review boards, following local requirements. This analysis includes the data used in the exploratory study that were collected between 2010 and 2016, and additional data collected between 2019 and 2020. Details about the project methodology were published previously (Bauer et al. 2012, 2014,2017).

### Data collection sites

Researchers from 71 collection sites in 40 countries provided the data, including those at university medical centers, specialty clinics and individual practitioners. Collection sites located in the northern hemisphere were: Aalborg, Denmark; Aarhus, Denmark; Ankara, Turkey; Athens, Greece; Bangkok, Thailand; Barcelona, Spain; Barhir Dar, Ethiopia; Beer Sheva, Israel; Belgrade, Serbia; Bengaluru, India; Cagliari, Sardinia, Italy (2 sites); Calgary, Canada; Dresden, Germany; Dublin, Ireland; Frankfurt, Germany; Halifax, Canada; Helsinki, Finland; Glasgow, UK; Gothenburg, Sweden; Grand Rapids, MI, USA; Hong Kong, China; Hyderabad, India; Iowa City, Iowa, USA; Jincheon, South Korea; Kampala, Uganda; Kansas City, KS, USA; Khanti-Mansiysk, Russia; Konya, Turkey; Kuala Lumpur, Malaysia; Los Angeles, CA, USA; Medellín, Colombia; Mexico City, Mexico; Milan, Italy; Oslo, Norway; Ottawa, Canada; Piacenza, Italy; Palo Alto, CA, USA; Paris, France (2 sites); Poznan, Poland; Rochester, MN, USA; Rome, Italy; San Diego, CA, USA; Siena, Italy; Singapore; Stockholm, Sweden; Tartu, Estonia; Thessaloniki, Greece (2 sites); Tokyo, Japan (3 sites); Taichung, Taiwan; Trondheim, Norway; Tunis, Tunisia; Vitoria, Spain; Wardha, India; Wiener Neustadt, Austria; Worcester, MA, USA, and Würzburg, Germany. Collection sites located in the southern hemisphere were: Adelaide, Australia; Melbourne/Geelong, Australia; Buenos Aires, Argentina; Cape Town, South Africa; Christchurch, New Zealand; Mataram, Indonesia; Porto Alegre, Brazil; Salvador, Brazil; Santiago, Chile (2 sites); and São Paulo, Brazil.

### Patient data collected

To facilitate international participation, minimal clinical data were collected for each patient. The patient data collected included gender, age of onset, polarity of first episode, family history of mood disorders, history of psychosis, episode course, history of alcohol and substance abuse, and history of suicide attempts. Three locations were also collected for each patient: birth location, onset location and current location. The same birth cohort groups were used as in the exploratory analysis, and in prior research (Bauer et al. 2014, 2015, 2017; Chengappa et al. 2003).

### Country specific data

Country specific socioeconomic data were obtained for all onset locations, including physician density per 1000 population, country median age, unemployment rate, poverty rate, gross domestic product (GDP) per capita (CIA World Factbook 2020), psychiatrists per 100,000 (WHO 2019a), Gini index of income inequality, percent Internet users (World Bank 2020a, b), gender inequality index (UN 2020), and if the country has a state-sponsored or officially favored religion (Pew Research 2017).

### Solar insolation

The NASA POWER database provides average monthly solar insolation expressed in kilowatt hours/square meter/day (kWh/m^2^/day) based on satellite observations collected between 1983 and the present (Stackhouse et al. 2018; NASA 2020). As in the exploratory study, a 22-year climatology of insolation spanning Jan 1984–December 2013 at spatial resolution of 1º × 1º latitude/longitude was used in this analysis. The actual onset locations were grouped into reference onset locations representing all onset locations within a 1º × 1º grid of latitude and longitude. For example, Dresden, Germany at latitude of 51.1° north and 13.8° east is the reference onset location for all locations between 51° and 52° north, and 13° and 14° east. The latitude and longitude of the reference onset location were used to identify solar insolation values for each patient.

During a year, the pattern of mean monthly solar insolation varies by latitude, with little change near the equator and large changes near the north and south poles. Solar insolation values for locations at the same latitude may vary significantly due to local conditions including cloud cover, aerosols (including dust and pollution), water vapor amounts, and altitude. Locations in the tropics (less than 23.5° north or south of the equator), may have a wet season where clouds reduce solar insolation and a dry season with clear skies rather than a winter/summer insolation pattern. To summarize the changes in solar insolation throughout the year at each reference onset location, two variables were created: (1) the ratio of the mean northern hemisphere winter (December, January, February) to the mean summer (June, July, August) insolation, and (2) the ratio of the minimum mean monthly insolation to the maximum mean monthly insolation. The insolation data from the southern hemisphere were shifted by 6 months for comparison to data from the northern hemisphere to account for the seasonal cycle.

### Statistics

The same statistical approach was used as in the exploratory study (Bauer et al. 2019), in which the generalized estimating equations (GEE) statistical technique was used to account for both the correlated data and unbalanced number of patients at reference onset locations. The GEE technique estimates the dependent variable as a function of the entire population, producing a population averaged or marginal estimates of model coefficients (Zeger and Liang 1986). All GEE models in this study were estimated using a binomial distribution, an exchangeable working correlation matrix and a logit link function where a patient history of suicide attempts was the dependent binary variable. The selection process of the best model of a history of suicide attempts first identified individual independent variables with an estimated coefficient significant at the 0.05 level in a univariate GEE model. Significant independent variables from univariate models and variables found in prior suicide research were then combined into multivariate GEE models of a history of suicide attempts. To identify the best model, the multivariate model estimates were compared using the corrected quasi-likelihood independence model criterion (Pan 2001) and confidence intervals at the 0.01 significance level to reduce the chance of type 1 errors. Based on the logit link function, the exponential coefficient can be interpreted as the effect size (Li et al. 2019). Demographic variables were reported using descriptive statistics. SPSS version 26.0 was used for all analyses.

## Results

### Available data

Data for 10,771 patients were available from the 71 collection sites, including 3379 new patients, 46% more patients than in the exploratory analysis. Of these, 7844 patients had a diagnosis of bipolar I disorder. Of the 7844 patients with bipolar I disorder, a history of suicide attempts was available for 6064 patients. Of the 6064 patients with data on a history of suicide attempts, all 5 variables in the best model were only available for 4876 patients, with 81% of the excluded patients missing data for a history of alcohol or substance abuse. Although 19.6% of the patients with data on a history of suicide attempts were excluded, the other demographics were similar to those included in the best model. The demographics of the 4876 patients included in the best model are shown in Table 1. Of the 4876 patients, 2760 patients (56.6%) were female, and 1496 patients (30.7%) had a history of suicide attempts.

**Table 1:** Demographics of the patients with bipolar I disorder (N = 4876)

Parameter	Value	N	%
Gender			
	Female	2760	56.6
	Male	2116	43.4
Polarity of first episode^a^			
	Manic/hypomanic	2302	48.8
	Depressed	2419	51.2
Family history of mood disorder^a^			
	No	2026	45.3
	Yes	2448	54.7
Alcohol or substance abuse			
	No	3369	69.1
	Yes	1507	30.9
State sponsored religion in country of onset			
	No	2662	54.6
	Yes	2214	45.4
History of suicide attempt			
	No	3380	69.3
	Yes	1496	30.7
Cohort group			
	DOB < 1940	179	3.7
	DOB ≥ 1940 and DOB < 1960	1241	25.5
	DOB ≥ 1960	3456	70.9

Parameter		Mean	SD
Age at time of data collection		47.8	14.4
Age of onset		25.7	10.6

^a^Missing values excluded

### Onset locations

**Fig. 1:**
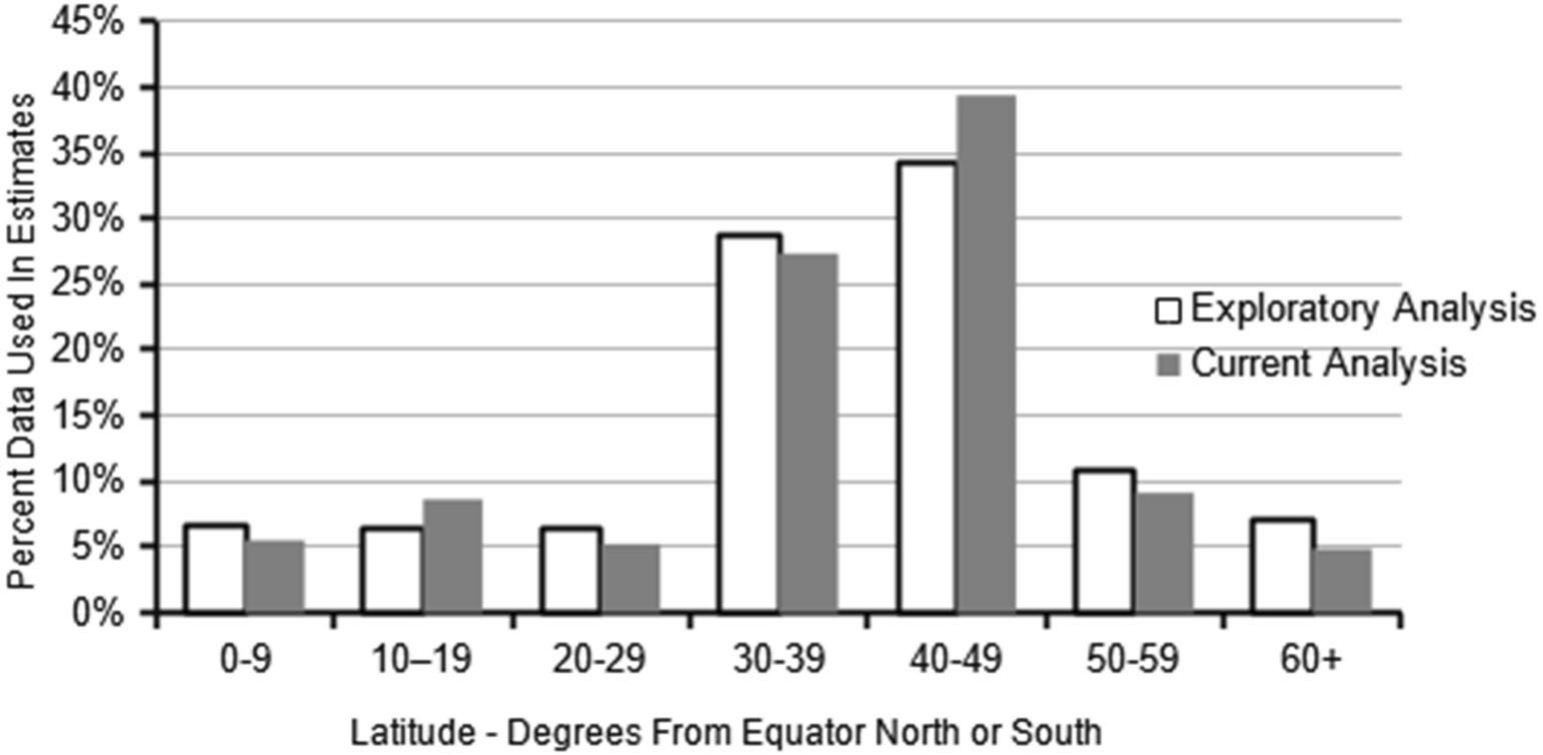
Comparison of range of onset location latitudes for current and exploratory analyses for patients with bipolar I disorder (N = 4876)

For the 4876 patients analyzed in the best model, there were 479 reference onset locations in 64 countries. The onset location was in the northern hemisphere for 4176 patients (85.6%), and in the southern hemisphere for 700 patients (14.4%), similar to estimates that about 12.5% of the world population lives in the southern hemisphere (Kummu and Varis 2011). Of the 4876 patients, 912 (18.7%) had an onset location in the tropics. For the 4876 patients, 97.6% of the onset locations were in the same country as the current country, and 83.3% of the onset cities were the same as the current city. The average number of patients in each onset location was 10.2, with 256 (5.3%) of the 4876 patients in an onset location with a single patient. As with the exploratory study, the much larger number of onset locations than collection sites reflects worldwide urbanization (WHO 2019b). Figure 1 provides a comparison of the range of latitudes for the onset locations between this analysis and the exploratory study.

### Ratio of mean winter solar insolation to mean summer solar insolation

At locations near the equator, there is little change in solar insolation between winter and summer and the ratio of mean winter solar insolation to mean summer solar insolation is large (near 1). At locations near the poles, solar insolation is very small in winter when compared to the summer, and the ratio is small (near 0). The ratio of mean winter solar insolation to mean summer solar insolation by latitude groups is shown in Table 2. The ratio of mean winter solar insolation to mean summer solar insolation for example onset locations in the latitude groups is shown in Table 3.

**Table 2:** Ratio of mean winter solar insolation/mean summer solar insolation and ratio of minimum mean monthly insolation/maximum mean monthly insolation by latitude for patient onset locations (N = 4876)

Degrees latitude north + south	N	%	Ratio mean winter insolation/mean summer insolation	Ratio minimum mean monthly insolation/maximum mean monthly insolation
0–9	268	5.5	1.0313	0.8076
10–19	420	8.6	1.1074	0.6744
20–29	254	5.2	0.7772	0.6093
30–39	1333	27.3	0.4075	0.3165
40–49	1921	39.4	0.3023	0.2119
50–59	444	9.1	0.1662	0.0903
60 +	236	4.8	0.0857	0.0220
Total	4876	100.0	0.4423	0.3135

**Table 3:** Ratio of mean winter solar insolation/mean summer solar insolation and ratio of minimum mean monthly insolation/maximum mean monthly insolation: example onset locations by latitude group (N = 4876)

Degrees latitude north + south	Onset location	Ratio mean winter insolation/mean summer insolation	Ratio minimum mean monthly insolation/maximum mean monthly insolation
0–9	Kampala, Uganda Kuala Lumpur, Malaysia Mataram, Indonesia Medellín, Columbia Singapore	1.1400 0.9702 1.0125 0.9065 1.0560	0.8197 0.7694 0.7831 0.8370 0.7797
10–19	Bahir Dar, Ethiopia Bangkok, Thailand Bengaluru, India Hyderabad, India Mexico City, Mexico Salvador, Brazil	1.1639 1.0680 1.1702 1.1762 0.9074 0.6844	0.7713 0.7207 0.6814 0.6421 0.6855 0.6246
20–29	Hong Kong, China São Paulo, Brazil Taichung, Taiwan Wardha, India	0.6603 0.7419 0.4492 1.1545	0.6016 0.6050 0.3931 0.5750
30–39	Ankara, Turkey Athens, Greece Beer Sheva, Israel Buenos Aires, Argentina Cagliari, Italy Cape Town, South Africa Los Angeles, CA, USA Melbourne, Australia San Francisco, CA, USA Santiago, Chile Seoul, South Korea Tokyo, Japan Tunis, Tunisia	0.3266 0.3148 0.4246 0.3978 0.3066 0.3873 0.4235 0.3628 0.4163 0.3537 0.6406 0.7201 0.3695	0.2374 0.2319 0.3556 0.3149 0.2328 0.3227 0.3503 0.2913 0.3137 0.2879 0.4404 0.5574 0.2859
40–49	Belgrade, Serbia Barcelona, Spain Boston, MA, USA Christchurch, New Zealand Grand Rapids, MI, USA Halifax, Canada Minneapolis, MN, USA Paris, France Rome, Italy Siena, Italy Vienna, Austria Würzburg, Germany	0.2832 0.3622 0.3626 0.3225 0.3281 0.3300 0.3339 0.2317 0.2993 0.2988 0.2631 0.2381	0.1960 0.2603 0.2662 0.2461 0.2256 0.2270 0.2371 0.1540 0.2203 0.2077 0.1667 0.1477
50–59	Aarhus, Denmark Calgary, Canada Dresden, Germany Dublin, Ireland Oslo, Norway Poznan, Poland Stockholm, Sweden Tartu, Estonia	0.1432 0.2269 0.2255 0.1927 0.1126 0.2127 0.1087 0.1353	0.0782 0.1454 0.1379 0.1149 0.0433 0.1290 0.0427 0.0562
60 +	Helsinki, Finland Khanti-Mansiysk, Russia Trondheim, Norway	0.1095 0.0951 0.0673	0.0359 0.0243 0.0116

### Best model results

The best fitting model for a history of suicide attempts uses the ratio of mean winter solar insolation to the mean summer solar insolation and is shown in Table 4. This is the same model that was selected in the exploratory study as the best model, and the estimated coefficients are similar in value to those in the exploratory analysis. The inclusion of 4876 patients in the best model was a 45% increase over the 3365 patients included in the exploratory analysis.

**Table 4:** Estimated parameters for best model explaining a history of suicide attempts for patients with bipolar I disorder (N = 4876)

Parameters				99% Confidence interval		Coefficient significance	
	Coefficient estimate (β)	Standard error	Exp (β)	Lower	Upper	Wald Chi-squared	P
Intercept	− 0.935	0.2279	0.393	− 1.522	− 0.348	16.815	< 0.001
Ratio mean winter insolation/mean summer insolation	− 0.730	0.1752	0.482	− 1.181	− 0.279	17.357	< 0.001
State sponsored religion in onset country	− 0.438	0.1145	0.645	− 0.733	− 0.143	14.655	< 0.001
Male	− 0.609	0.0792	0.544	− 0.813	− 0.405	59.096	< 0.001
History of alcohol or substance abuse	0.459	0.0726	1.582	0.272	0.646	39.978	< 0.001
DOB ≥ 1960	0.822	0.2289	2.275	0.232	1.414	12.890	< 0.001^a^
DOB ≥ 1940 and DOB < 1960	0.681	0.2064	1.975	0.149	1.212	10.872	0.001^a^

Dependent variable: history of suicide attempts (yes/no). Model: intercept, ratio of mean winter insolation/mean summer insolation at onset location, gender, state sponsored religion in onset country (yes/no), alcohol or substance abuse (yes/no) and birth cohort group (DOB < 1940, DOB ≥ 1940 and DOB < 1960, DOB ≥ 1960)

^a^Individual parameters Wald Chi-square statistics and significance. The model effects Wald Chi-square and significance for the cohort parameter was 12.904 and 0.002, respectively with 2 degrees of freedom

The estimated coefficients for the model suggest that the odds of a suicide attempt will decrease by 4.8% for every 0.1 increase in the ratio of mean winter to summer insolation. Alternatively stated, comparing a ratio of 1 (near the equator) to a ratio of 0 (near a pole), there was a 48% difference in the odds of a suicide attempt with the lowest odds at the equator. The model estimates that being male will decrease the odds of a suicide attempt by 54%, and living in a country with a state sponsored or favored religion will decrease the odds by 65%. The model also estimates that having a history of alcohol or substance abuse will increase the odds of a suicide attempt by 58%, and being in the youngest cohort will increase the odds of a suicide attempt by 127%.

### Ratio of minimum mean monthly insolation to the maximum mean monthly insolation

The ratio of minimum mean monthly insolation to the maximum mean monthly insolation by latitude groups is shown in Table 2. The ratio of minimum mean monthly insolation to the maximum mean monthly insolation for selected onset locations in the latitude groups is shown in Table 3. A second model that substituted the ratio of minimum mean monthly insolation to the maximum mean monthly insolation for the ratio of mean winter solar insolation to mean summer solar insolation was estimated using the same data as with the best model.

The estimated coefficients for the model using the minimum mean monthly insolation to the maximum mean monthly insolation are shown in Table 5, and are very similar to those in the best model. The estimated coefficients for the monthly model suggest that the odds of a suicide attempt will decrease by 4.4% for every 0.1 increase in the ratio of mean winter to summer insolation. Alternatively stated, comparing a ratio of 1 (near the equator) to a ratio of 0 (near a pole), there was a 44% difference in the odds of a suicide attempt with the lowest odds at the equator. The model estimates that being male will decrease the odds of a suicide attempt by 54%, and living in a country with a state sponsored or favored religion will decrease the odds by 69%. The model also estimates that having a history of alcohol or substance will increase the odds of a suicide attempt by 59%, and being in the youngest cohort will increase the odds of a suicide attempt by 124%.

**Table 5:** Estimated parameters for alternative model explaining a history of suicide attempts for patients with bipolar I disorder (N = 4876)

Parameters				99% Confidence interval		Coefficient significance	
	Coefficient estimate (β)	Standard error	Exp (β)	Lower	Upper	Wald Chi-squared	P
Intercept	− 1.026	0.2302	0.358	− 1.619	− 0.434	19.885	< 0.001
Ratio minimum mean monthly insolation/maximum mean monthly insolation	− 0.813	0.2552	0.444	− 1.470	− 0.155	10.136	0.001
State sponsored religion in onset country	− 0.378	0.1127	0.685	− 0.668	− 0.088	11.252	0.001
Male	− 0.612	0.0794	0.542	− 0.816	− 0.407	59.438	< 0.001
History of alcohol or substance abuse	0.466	0.0730	1.594	0.278	0.655	40.760	< 0.001
DOB ≥ 1960	0.808	0.2312	2.244	0.213	1.404	12.224	< 0.001^a^
DOB ≥ 1940 and DOB < 1960	0.679	0.2085	1.972	0.142	1.216	10.612	0.001^a^

Dependent variable: history of suicide attempts (yes/no). Model: intercept, ratio minimum mean monthly insolation/maximum mean monthly insolation at onset location, gender, state sponsored religion in onset country (yes/no), alcohol or substance abuse (yes/no) and birth cohort group (DOB < 1940, DOB ≥ 1940 and DOB < 1960, DOB ≥ 1960)

^a^Individual parameters Wald Chi-square statistics and significance. The model effects Wald chi-square and significance for the cohort parameter was 12.224 and 0.002, respectively with 2 degrees of freedom

The collection site was thought to be an adequate proxy for the onset location for some or all patients from Barcelona, Cape Town, Christchurch, Frankfurt, Helsinki, Melbourne/Geelong, Porto Alegro, São Paulo, Salvador, Vitoria, and Würzburg, where the patient onset location was not provided. To test the effect of using the current location as a proxy for the onset location, the best model and the minimum mean monthly insolation to the maximum mean monthly insolation model were also estimated excluding these patients. The magnitude of the estimated coefficients did not change substantially and remained significant at the 0.01 level. Estimated models including other patient, country and solar insolation variables were not as significant, or not as meaningful.

## Discussion

This analysis confirmed the results of the exploratory study after including 45% more international patient data. Living in locations with a large change in solar insolation between winter and summer was associated with increased history of suicide attempts in patients with bipolar I disorder. The onset locations in this analysis were distributed across all latitudes in both hemispheres, and represent a wide range of solar insolation profiles and climatic conditions. The exploratory study results were confirmed in this study in two ways: by identifying the same GEE model as the best model, and by estimating a nearly identical relationship between solar insolation and a history of suicide attempts with slightly better statistical significance. In addition, the estimated coefficients for all other contributing variables in the model, history of alcohol or substance abuse, female gender, birth cohort and state sponsored religion, were similar and slightly more significant. The finding of nearly identical results with an alternative measure of variation in solar insolation, which applies to all locations including the tropics, further confirms the association between a change in solar insolation and a history of suicide attempts.

The largest change in solar insolation between winter and summer occurs at locations near the poles. Suicide is a serious public health problem in the 8 countries with Arctic communities above 60°N (Pollock et al. 2020; Young et al. 2015). For example, in 2017 the suicide rate for the state of Alaska was nearly double the US national suicide rate, and nearly triple for Alaska native people (AK-IBIS 2019). Additionally, seasonality in suicide is associated with latitude, with little monthly variation or seasonality in suicide rates near the equator, and spring and summer peaks in suicide rates with increasing latitudes north or south (Davis and Lowell 2002; Schwartz 2019).

There is related evidence involving patterns of solar radiation from studies within individual countries. In Finland, an increased suicide risk was associated with the cumulative low solar radiation over the long northern winter (Ruuhela et al. 2009). Several studies reported that an increasing risk of suicidal behavior was associated with increasing solar radiation. In South Korea, increased solar radiation in spring and summer was associated with an increased suicide rate (Jee et al. 2017). In Germany and Greece, increased solar insolation may precede suicidal acts (Müller et al. 2011; Papadopoulos et al. 2005). In Italy, higher solar radiation was associated with an increase in patients admitted to an emergency psychiatric unit with a primary diagnosis of bipolar disorder (Aguglia et al. 2019).

### Consistency with prior research

The demographics of the patients are consistent with prior international studies of bipolar disorder, with 30.7% having a history of suicide attempts (Tondo et al. 2016; Dong et al. 2019; Bobo et al. 2018), and 30.9% a history of alcohol or substance abuse (Toftdahl et al. 2016; Hunt et al. 2016; Grant et al. 2004; Nesvåg et al. 2015). Although we previously found a strong, inverse relation between the maximum monthly increase in solar insolation in springtime and the age of onset of bipolar I disorder (Bauer et al. 2017, 2014, 2012), the unadjusted mean age of onset of 25.7 is also similar to international studies (Baldessarini et al. 2012; Morselli et al. 2003; Kalman et al. 2019).

The other variables included in the best model also agree with prior suicide research in bipolar disorder and the general population. Alcohol and substance abuse (Schaffer et al. 2015; Carrà et al. 2014; Østergaard et al. 2017; Bobo 2018; Yuodelis-Flores and Ries 2015; Norström and Rossow 2016), and being female (Schaffer et al. 2015, Dong et al. 2019; Tondo et al. 2016; Bobo 2018) are associated with an increased risk of suicidal behavior. Increased suicide attempts or deaths are reported internationally in younger birth cohorts (Twenge et al. 2019; Odagiri et al. 2011; Page et al. 2013; Yu and Chen 2019; Kwon et al. 2009; Gunnell et al. 2003; Phillips 2014; Chung et al. 2016). Studies involving all major world religions find that religion may be protective against suicidal behavior (Eskin et al. 2020; Wu et al. 2015; VanderWeele et al. 2016; Stack and Kposowa 2011; Dervic et al. 2011; Caribe et al. 2015; Jacob et al. 2019).

### Special importance of daylight

The findings of this study highlight the importance of daylight to human wellbeing and behavior. In repeated surveys, people preferred daylight over electric lighting as the source of illumination, although the reasons for the strong daylight preference are not fully established (Knoop et al. 2020; Boyce et al. 2003; Haans 2014). Daylight differs from electric lighting in many fundamental properties, including the spectrum, intensity, temporal characteristics, flicker, and polarization, and the properties of daylight change throughout the day, month and year (Knoop et al. 2020; Aaarts et al. 2017). Many additional factors influence the physiological effects of light. These include individual characteristics such as age, lifestyle, health status, and genetics, environmental issues such as the season, climate, latitude and building design, and the duration of exposure and prior light exposure (Münch et al. 2017; 2020; Turner and Mainster 2008; Prayag et al. 2019).

Researchers emphasize the need to better understand how people respond to daylight and electric lighting in real-life settings (Knoop et al. 2020; Webler et al. 2019; Münch et al. 2020; Foster et al. 2020). Knowledge of non-image forming visual functions including circadian entrainment has grown rapidly. However, many findings are from small studies of healthy young adults exposed to electric lighting in controlled settings, or from animal studies. Even in controlled settings, considerable individual variability in sensitivity to light was detected (Phillips et al. 2019; McGlashan et al. 2018; Chellepa 2020). Understanding of how light intensity and duration of exposure interact for circadian entrainment is limited (Foster et al. 2020). Studies are needed that measure naturally occurring entrainment in large numbers of people of all ages and occupations, including mixed exposure to daylight and electric lighting in the day as well as electric lighting at night (Knoop et al. 2020; Webler et al. 2019; Münch et al. 2020; Foster et al. 2020). It is also not clear how applicable these findings are to patients with bipolar disorder. The optimal mix of daylight and electric lighting for circadian entrainment needs to be clarified to increase understanding of bipolar disorder and suicide risk, and improve the efficacy of chronotherapeutic treatments (Geoffroy and Palagini 2021; Gottlieb et al. 2019; Münch et al. 2020; Wang et al. 2020; Wirz-Justice and Benedetti 2020).

### Limitations

Data in this project were collected as a convenience sample. The diagnosis was based on DSM-IV or DSM-5 criteria, but data collection methods and sources were not standardized, including the definition of suicide attempts. Although convenience samples can contain inadvertent biases, this study repeated the results of the exploratory study using substantially more international patient data. This suggests either sample biases in the exploratory study were duplicated in the data collection for this study from 71 international collection sites or, more likely, the relationship found between solar insolation and a history of suicide attempts was confirmed.

Although a large percentage of patients had the same onset city and current city (83.3%), and the same onset and current country (97.6%), there was no confirmation that the suicide attempt occurred at the onset location. There was no data on individual risk factors for suicide attempts, the phase of bipolar disorder when the suicide attempt occurred, or treatments received for bipolar disorder, including those that may lower the risk of suicide such as lithium. There was no data on suicide deaths. The risks for attempted versus completed suicides could not be analyzed, although there are known distinctions (Hansson et al. 2018; Nock et al. 2008). There was no data on individuals who did not seek treatment. There was no individual data on sun exposure, sun-related activities, or lifestyle issues such as shift work. This analysis does not demonstrate causality or predict individual behavior. Characteristics of the forms of electric lighting were not discussed, and other environmental variables were not included. Data from the southern hemisphere were shifted by 6 months, disregarding cultural dimensions of seasonality. Religious and cultural differences may influence data collection related to suicide, and to alcohol and drug abuse. The premature mortality from general medical illness (Roshanaei-Moghaddam and Katon 2009), completed suicides, treatment dropout rates, and the increased rate of diagnosis of bipolar disorder over time (Blader and Carlson 2007) may bias findings related to the birth cohort.

We previously noted two issues related to solar insolation that should be investigated in relation to suicide attempts: the potential impacts of continuous low solar insolation in areas near the poles with winters that last longer than 3 months, and of regional variance in insolation that has occurred over decadal timeframes (Wild 2012). However, we felt it was important to first confirm the results of the exploratory study.

## Conclusion

A history of suicide attempts in patients with bipolar I disorder was associated with living in locations with a large change in solar insolation, both between winter and summer and between the minimum and maximum monthly values. Given the frequent presence of circadian rhythm dysfunction and suicidal behavior in bipolar disorder, and the fundamental importance of daylight to human health, greater understanding of the optimal roles of daylight and electric lighting in circadian entrainment in both the normal population and bipolar disorder is needed.

## Data Availability

The data will not be shared or made publicly available.
